# Polyglot Programming in Applications Used for Genetic Data Analysis

**DOI:** 10.1155/2014/253013

**Published:** 2014-08-14

**Authors:** Robert M. Nowak

**Affiliations:** Institute of Electronic Systems, Warsaw University of Technology, Nowowiejska 15/19, 00-665 Warsaw, Poland

## Abstract

Applications used for the analysis of genetic data process large volumes of data with complex algorithms. High performance, flexibility, and a user interface with a web browser are required by these solutions, which can be achieved by using multiple programming languages. In this study, I developed a freely available framework for building software to analyze genetic data, which uses C++, Python, JavaScript, and several libraries. This system was used to build a number of genetic data processing applications and it reduced the time and costs of development.

## 1. Background

The number of computer programs for the analysis of genetic data is increasing significantly, but it still needs to be improved greatly because of the importance of result analysis with appropriate methods and the exponential growth in the volume of genetic data.

Genetic data are typically represented by a set of strings [1], where each string is a sequence of symbols from a given alphabet. The string representation, called primary structure, reflects the fact that the molecules storing genetic information (DNA and RNA) are biopolymers of nucleotides, while proteins are polypeptide chains. The secondary, tertiary, and quaternary structures need to be considered to understand the interactions among nucleotides or amino acids, but they are used less frequently in computer programs. The secondary structure includes the hydrogen bonds between nucleotides in DNA and RNA and the hydrogen bonds between peptide groups in proteins, where the molecules are represented by graphs. The tertiary structure refers to the positions of atoms in three-dimensional space, and the quaternary structure represents the higher level of organization of molecules. The representations of molecules are extended based on connections between sequences or subsequences, which denotes similarity from various perspectives. Moreover, these data are supplemented with human-readable descriptions, which facilitate an understanding of the biological meanings of the sequence, that is, its function and/or its structure.

The large number of possible candidate solutions during the analysis of genetic data means that the employed algorithms must be selected carefully [2]. Exhaustive search algorithms must be supported by heuristics based on biological properties of the modeled objects. Of particular importance in this field are dynamic programming algorithms, which allow us to find the optimal alignment of biological sequences (i.e., arranging the sequences by inserting gaps to identify regions of similarity [1]) in polynomial time, although the search space grows exponentially. Dynamic programming is used to search for similarity (local or global), to generate a multisequence representation (profile), and to examine sequences with hidden Markov models. In addition, backtracking algorithms are used to search for motifs (i.e., identifying meaningful patterns in genetic sequences), greedy algorithms to detect genome rearrangements and to sort by reversals, divide-and-conquer algorithms to perform space-efficient sequence alignments, and graph algorithms for DNA assembly.

A characteristic feature of the computer programs applied to genetic data is the necessity to analyze large amounts of data using complex algorithms, which means that high performance is crucial. Different user and system requirements mean that the flexibility of software is also important. Finally, users prefer a graphical interface that is accessible from a web browser and applications that update automatically.

Scientists are becoming increasingly involved in software development [3]. They should use software engineering practices and tools to avoid common mistakes and to speed up the development tasks [4]. The architecture of working application with explanation of development decisions could help in developing new computer programs. Biological and medical terminology is simplified to invite developers to discuss the presented solutions.

In this study, I describe the* bioweb* framework, including application architecture, the programming languages, libraries, and tools, used to develop applications for processing genetic data. I propose a multilanguage platform using C++, Python, and JavaScript. The use of appropriate and tested architectures, libraries, and tools decreases the risk of failure in software system development as well as reduces the costs and time requirements. The use of appropriate systems also facilitates rapid prototyping, which allows us to verify concepts by obtaining the requisite information from end users: biologists and doctors.

## 2. Results

### 2.1. Deployment Model

A three-layer software architecture was selected where the presentation layer, data processing layer, and data storage layer were kept separate. The use of a multilayered model makes computer programs flexible and reusable, because applications have different responsibilities. Thus, it is beneficial to segregate models into layers that communicate via well-defined interfaces. Layers help to separate different subsystems, and the code is easier to maintain, clean, and well structured.

**Figure 1:**
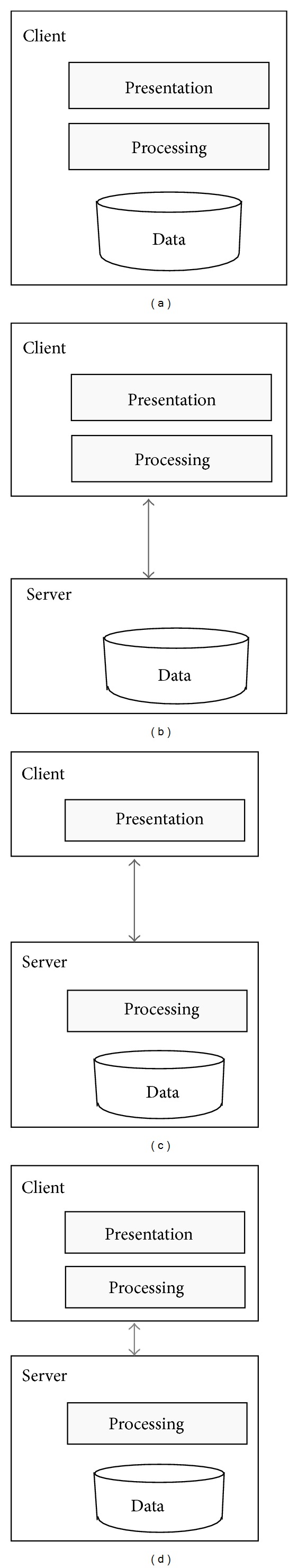
Three-layer application deployment models: desktop application (a), database server (b), thin client (c), and web application (d). This solution supports the creation of applications using a web application architecture.

Four possible deployment models were considered for the three-layer architecture: the desktop, the database server, the thin client, and the web application, as shown in Figure 1. The desktop architecture (Figure 1(a)) was rejected because the framework was designed to support multiuser applications. Collaboration features were hard to implement in this architecture because of the lack of central data server that could be accessed by multiple users. The offline mode is rarely used because the Internet is available almost everywhere and the transmission costs are negligible compared with the costs of maintaining the system. Furthermore, sequence databases are publicly available via the Internet, so an Internet connection is essential for the analysis of genetic data.

An application architecture with a shared database and data processing modules deployed on client machine (Figure 1(b)) was rejected because of the requirement for high client computer performance. Another problem is the need to update the software on the client side when changes and additions are made, which is time consuming and requires support for a wide range of platforms so the development costs are high.

Deploying the calculation modules on a server machine allows the execution of these modules by clients on different platforms, which reduces the development costs. The computational power of the server is important because it determines the computational time, which means that poorly equipped client machines can be used. The optimum solutions are a thin client architecture, as shown in Figure 1(c), and a web application architecture, as shown in Figure 1(d).

Deploying the calculation modules on a server machine, as shown in Figures 1(c) and 1(d), allows the use of many platforms on the client side, which reduces the development costs. Importantly, the computational power of the server is used, so the computational time can be relatively short, even for poorly equipped client machines. These solutions simplify scalability if the size of the problem or the number of clients grows, because only the servers need to be upgraded.

Web applications have advantages compared with application produced with a thin client architecture because the client contains a portion of the data processing layer, which can handle activities such as output reformatting, graph generation, and user input validation. Client-based processing reduces the amount and frequency of client-server traffic, and it reduces the load on the server while the reactions to user actions are faster. This solution uses web browser plugins (such as Flash) or HTML5/JavaScript programs on the client side. The client modules are downloaded during initialization, which helps to avoid the issue of updating the software.

### 2.2. Architecture and Programming Languages

**Figure 2:**
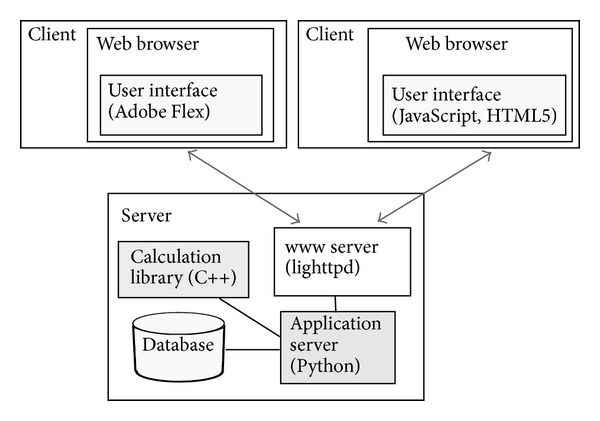
Modules produced for a typical application based on the proposed framework using various programming languages.

The software used by presented framework and the framework itself were created with C++, Python, and JavaScript with HTML5. The use of multiple languages in a single project is quite common and it is an alternative to using PHP, NET, or Java. The set of used languages facilitates high performance, versatility, customizable modules, and the production of a web browser interface. The modules produced for a typical application based on* bioweb* using these programming languages are shown in Figure 2.

The algorithms are implemented in C++. The source code is translated (compiled) into machine language, which makes algorithm execution more efficient because the code is executed directly by the processor. The language has higher-level abstractions missing in other languages translated into binary code (C and Fortran). C++ supports object-oriented programming by providing virtual functions and multibase inheritance and exceptions and facilitates functional and genetic programming, including templates and lambda functions. The standard C++ library is compact but it is well tested and efficient. It includes support for inputs and outputs, strings and string operations such as regular expressions, and sets of collections, such as vectors, lists, sets, and associative arrays using trees and/or hash tables. It should be mentioned that concurrency support mechanisms are included in the C++11 standard (ISO/IEC 14882:2011), so the full capabilities of modern computers with multiple processors and/or multiple cores can be exploited. If an older C++ compiler that does not support C++11 is used, it may be necessary to employ the Boost [5] libraries: Boost.Thread to create portable multithread applications, Boost.Regex for regular expressions, and Boost.Chrono for time utilities. In addition, vector calculations provided by modern graphics processing unit (GPU) are available in C++ and the OpenCL [6] standard is applied.

The server application uses the Python language in presented solution, mainly because this type of development is faster compared with C++. Modules that do not constitute a bottleneck during calculations should be implemented in Python. Python is a scripting language, so it is small and has a simple, regular syntax. This language is dynamically type-checked, uses a uniform data model, and provides reference counting memory management, so there is no problem with memory leaks. The Python repository of software (PIP) https://pypi.python.org/pypi contains over 30,000 packages and a number of ready-made solutions can be used, particularly the packages for exchanging data and packages that support the creation of the web applications I used. It should be noted that the Biopython library [7] provides a set of tools for biological computation which are written in Python.

In* bioweb* the Boost.Python [5] library enables interoperability between the C++ modules and the Python modules. Other solutions, such as using C API from Python directly, code generation using Simplified Wrapper and Interface Generator (SWIG), Py++, Pyrex, and cython, were considered to be less useful because the interface was less convenient and there was a lack of support for the techniques used in genetic data software development. The Boost.Python uses C Python API and metaprogramming techniques, which allows the exposure of C++ classes, functions, and objects to Python and vice-versa, thereby supporting the use of Python facilities inside C++ code. Boost.Python allows the exposure of elements and the register of conversions using a simple syntax and being easy to learn.

The use of a compiler and an interpreter makes the developed software more flexible. The application customization requires the use of an interpreter in any case, because changing the settings should not demand the software rebuilding. The use of Python to store the user settings simplifies the customization of applications greatly, because the settings do not need to be lists of names and values, and the Python control instructions can be used.

A client application request is sent to the standard port using the HTTP protocol and it is retransmitted by the web server using interprocess communication mechanisms (e.g., sockets and named pipes) to the server application. Three web servers were investigated: Apache http://httpd.apache.org, Lighttpd http://www.lighttpd.net, and Nginx http://nginx.org. The Lighttpd configuration is known to be simple and its performance is very good, so the presented solution only includes settings for this web server, but* bioweb* is also able to use Apache and Nginx. So scripts available on project website only include a setting for this web server. Lighttpd retransmission uses mod_fastcgi and a socket mechanism. Three open source Python libraries were considered: Flup from PIP, Web2py http://www.web2py.com, and Django https://www.djangoproject.com. The libraries support the Web Server Gateway Interface (WSGI), the Python standard interface between web servers and applications.

Flup is a simple WSGI server but its library is small (256 kB), so the facilities are limited to the python function call when an http request is received from a client and the function results are sent back to the client application using a web server. More advanced libraries are Web2Py (9 MB) and Django (22 MB), where the facilities include parameter conversion, authentication, authorization, and database support using object-relational mapping. All Flup, Web2py, and Django were tested in the present study, because the characteristics of Web2py and Django are similar. However, Django is recommended because all of the available facilities are written explicitly and this library has the best documentation. Django uses Flup internally to cooperate with Lighttpd in current version of software; this configuration works correctly under all popular modern operating systems (Linux, Windows, iOS, etc.).

*Bioweb* provides two competitive solutions for client modules, where the first is based on JavaScript with HTML5, and the second uses Apache Flex and the Adobe Flash Player plugin. JavaScript with HTML5 web applications uses the Ajax techniques available on modern web browsers, mainly XMLHttpRequest objects, so client applications developed in JavaScript can send and retrieve data in the background. The data are interchanged using the JavaScript Object Notation (JSON), and the Python standard library module supports JSON encoders and decoders. The HTML5 standard includes scalable vector graphics support, which improves the graphical user interface. JavaScript is interpreted by a web browser and it conforms to international standard ISO/IEC 16262:2011. The current version uses Model View ViewModel (MVVM) client-side JavaScript framework AngularJS [8].

Apache Flex is a freely available set of software development tools, which support the construction of applications that use the Adobe Flash Player plugin. This plugin, which is available for most web browsers, allows the user to view multimedia, vector graphics, and animations. The Apache Flex application is loaded from web server and executed on the client side. Communication with the server uses the Action Message Format (AMF), which is supported in Python by the pyAMF library. At present, this technology is being replaced by HTML5, which is supported directly by web browsers, so HTML5 and JavaScript are recommended for use in new applications.

### 2.3. Parallel Service Requests

**Figure 3:**
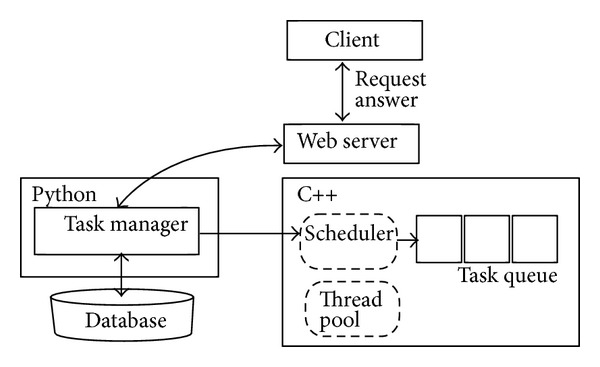
Active object implementation delivered by the framework. The client requests are transformed into commands automatically, which are executed by separate threads.

The framework was designed to create the software that serves multiple users at the same time. The users communicate independently with the server via the Internet and the framework includes a component with the active object pattern [9] implementation to enhance concurrency and to exploit the server resources fully. This component, which is part of* bioweb*, is shown in Figure 3.

**Figure 4:**
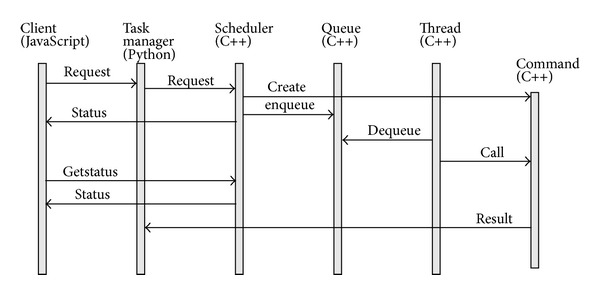
Cooperation among active object participants. The client request is converted into a command managed by the task manager on the Python side and by the scheduler in C++. The command is stored in the queue, and it is executed when an unoccupied thread is available. The client can request the current command status and the command progress.

The execution of calculation tasks is decoupled from task invocation to enhance concurrency and to simplify multithread usage, as shown in Figure 4. Calculation requests sent from the client application are converted into C++ objects. These objects are commands (the command design pattern is used) which contain specific parameters as well as algorithm and synchronization mechanisms. Commands are stored in the task queue and executed by separate execution threads from the thread pool. The command handlers are accessible from Python, so the user can examine the current command state, that is, tasks that are awaiting execution in the queue, executed tasks, and completed tasks. This component uses an observer (from observer design pattern), to support the command progress notification. The active object module can be used independently of* bioweb*; it is supplied separately as a C++ library, whose sources are available at http://mt4cpp.sourceforge.net.

### 2.4. Testing

Software testing is an integral part of the development process. Thus, testing techniques and libraries that support this process are specified in presented framework. Three types of tests are considered: unit tests, integration tests, and system tests. Unit testing checks individual functions, procedures, and classes in isolation. Integration tests examine the communication between modules, based on a consideration that they are created in different programming languages. System tests examine the functions of a computer program as a whole, without the knowledge of the internal structure of the software.

Unit testing uses Boost.Test [5] for C++ modules, the standard Python unittest package for Python code, and QUnit http://qunitjs.com for modules written in JavaScript. C++ unit testing is performed in both environments: g++ and msvc. Integration tests are implemented with the same tools and libraries as unit tests, but the features of C++ modules exported to Python by the Boost.Python library are tested in Python using unittest.

System testing uses the Python language and splinter http://splinter.cobrateam.info library. This tool automates browser actions such as visiting URLs, navigation, verifying page context, finding elements in the page, testing mouse and keyboard events, reading the text properties of elements, and other tasks. The system tests allow the automatic evaluation of test scenarios, without any requirement for manual testers, which reduces the time and the cost of the overall system examination.

The test quality measure is the source code coverage during unit, integration, and system testing. This measure provides numerical data related to the performance of test procedures, which helps to identify inadequately tested parts of the software. The analytic tools used to evaluate coverage in* bioweb* are gcov from the GNU Compiler Collection for C++ modules, Coverage.py from Python Package Index (PIP) for Python modules, and Blanket.js http://blanketjs.org/ for JavaScript code.

### 2.5. Tools

This section describes the programming tools used to create applications in* bioweb*. It is important that the latest versions of the tools described are used.

The C++ modules require a C++ compiler and it is recommended to use at least two different compilers, particularly the g++ compiler from the GNU Compiler Collection http://gcc.gnu.org and the Microsoft Visual C++ Compiler (msvc) http://msdn.microsoft.com. The use of different compilers increases the probability of capturing errors in the code and it ensures that the code is portable. The C++ modules use the standard C++ library and the Boost http://www.boost.org libraries. The server uses the Python interpreter, the Python standard library, and packages from the Python repository (PIP). The client uses the JavaScript interpreter built-in web and the AngularJS [8] framework, jQuery libraries http://jquery.com. The Bower [10] automatically manages client-side package dependencies. An alternative is to use the Apache Flex software developer's kit http://flex.apache.org. The Scons http://www.scons.org is used to create modules, for testing, and to consolidate the whole system, while Redmine http://www.redmine.org is used for project management, and mercurial http://mercurial.selenic.com is used as the version control system.

## 3. Discussion

To speed up the creation of new software, the developer can use a specialized framework. The most popular, freely available frameworks are Bioconductor [11], MEGA tool [12], and OpenMS [13]. On the other hand, the programmer can use general-purpose programming language and specialized libraries, for example, C++ with NCBI C++ Toolkit [14], Python with BioPython [7], Java with BioJava [15], and BioWeka [16]. All these solutions impose limitations connected with the usage of only one programming language [17] and do not support the user interface in a web browser.

The polyglot environment is common among web software, that is, software accessible from a web browser, because the client-side software (JavaScript, HTML, and CSS) has different responsibilities compared to server side. The ubiquity of mobile applications and the advent of big data change the software development to use multiple languages [18]. Similar trends are evident in the bioinformatics software and the examples are GBrowse [19] or GEMBASSY [20].* Bioweb* provides a framework for the construction of such applications.

There are many application development frameworks that connect C++ with Python or Python with JavaScript. Presented solution is similar but combines three programming languages.

The* bioweb* is small, but it can be extended, and it can use specialized libraries. The heavyweight web-based genome analysis frameworks, such as Galaxy [21], have a lot of ready-made modules and meet most of the requirements for systems for the genetic data analysis. However, creating custom modules and algorithms is not trivial. Presented framework allows the user to create smaller and independent solutions, which are easier to manage and to customize. It could be easily extended to use GPU and/or computing clusters, which is required in production-scale analysis.

## 4. Conclusion

The* bioweb* framework is freely available from http://bioweb.sourceforge.net under GNU Library or Lesser General Public License version 3.0 (LGPLv3). All of the libraries and applications used in* bioweb* are available for free and they can be used in commercial software.

This framework was used to create several applications to analyze genetic data:* DNASynth* application for synthesizing artificial genes (i.e., completely synthetic double-stranded DNA molecules coding peptide), the* DNAMarkers* application for analyzing DNA mixtures, the* CodonHmm* application for protein back-translation, the* WebOmicsViewer* application for storing and analyzing genomes, the* PETconn* application to create scaffolds using paired-end tags, and the* DNAAssembler* for assembling DNA using next-generation sequencing data. The source code for these applications is available on the project website.

This genetic data analysis software development project was performed in academia and it supports students who have a limited amount of time available and who also lack experience in design and programming. I found that agile methodologies [22] worked well in this project because they support the transfer of biological and medical knowledge from the users of the application. They let us avoid the duplication of information and allowed minimal documentation production, so a task could be completed relatively quickly by new users. In particular, the SCRUM [23] and the extreme programming (XP) [24] techniques were used, that is, SCRUM roles (product owner, development team, and scrum master), SCRUM iterations (sprint planning meeting, end meetings), SCRUM task management and prioritizing, XP test-driven development, and XP coding and documentation.

Presented framework is still being developed; the Guncorn [25] Python HTTP Server is added to the upcoming version. This cancels the Flup on Unix platforms and accelerates data transfer between client and server.

*Availability and Requirements*

- project homepage: http://bioweb.sourceforge.net;
- operating systems(s): OS Portable;
- programming language: C++ and Python and JavaScript;
- license: GNU Library or Lesser General Public License version 3.0 (LGPLv3);
- getting started: to build a “Hello World” application please download the latest version, extract the files from the archive, install additional software as described in  README_EN (text file in main bioweb directory), and run  scons command in the directory where you placed the bioweb. To start the client and server locally run scons  r=1.

## References

[1] Durbin R (1998). *Biological Sequence Analysis: Probabilistic Models of Proteins and Nucleic Acids*.

[2] Jones NC, Pevzner P (2004). *An Introduction to Bioinformatics Algorithms*.

[3] Osborne JM, Bernabeu MO, Bruna M (2014). Ten simple rules for effective computational research. *PLoS Biology*.

[4] Wilson G, Aruliah D, Brown CT (2014). Best practices for scientific computing. *PLoS Biology*.

[5] Nowak R, Pajak A (2010). *C++ Language: mechanisms, design patterns, libraries*.

[6] Stone JE, Gohara D, Shi G (2010). OpenCL: a parallel programming standard for heterogeneous computing systems. *Computing in Science and Engineering*.

[7] Cock PJA, Antao T, Chang JT (2009). Biopython: freely available python tools for computational molecular biology and bioinformatics. *Bioinformatics*.

[8] Darwin PB, Kozlowski P (2013). *AngularJS Web Application Development*.

[9] Lavender RG, Schmidt DC Active object—an object behavioral pattern for concurrent programming.

[10] http://bower.io/.

[11] Gentleman RC, Carey VJ, Bates DM (2004). Bioconductor: open software development for computational biology and bioinformatics. *Genome Biology*.

[12] Kumar S, Tamura K, Nei M (2004). MEGA3: integrated software for molecular evolutionary genetics analysis and sequence alignment. *Briefings in Bioinformatics*.

[13] Sturm M, Bertsch A, Gröpl C (2008). OpenMS—an open-source software framework for mass spectrometry. *BMC Bioinformatics*.

[14] Vakatov D

[15] Holland RCG, Down TA, Pocock M (2008). BioJava: an open-source framework for bioinformatics. *Bioinformatics*.

[16] Gewehr JE, Szugat M, Zimmer R (2007). BioWeka: extending the Weka framework for bioinformatics. *Bioinformatics*.

[17] Fourment M, Gillings MR (2008). A comparison of common programming languages used in bioinformatics. *BMC Bioinformatics*.

[18] Binstock A http://www.drdobbs.com/architecture-and-design/the-quiet-revolution-in-programming/240152206.

[19] Wilkinson M (2006). Gbrowse Moby: a Web-based browser for BioMoby services. *Source Code for Biology and Medicine*.

[20] Itaya H, Oshita K, Arakawa K, Tomita M (2013). GEMBASSY: an EMBOSS associated software package for comprehensive genome analyses. *Source Code for Biology and Medicine*.

[21] Goecks J, Nekrutenko A, Taylor J (2010). Galaxy: a comprehensive approach for supporting accessible, reproducible, and transparent computational research in the life sciences. *Genome Biology*.

[22] Fowler M, Highsmith J (2001). The agile manifesto. *Software Development*.

[23] Schwaber K (2004). *Agile Project Management with SCRUM*.

[24] Beck K, Andres C (2004). *Extreme Programming Explained: Embrace Change*.

[25] Gunicorn http://gunicorn.org/.

